# Supplementary material for: A random-walk-based epidemiological model
Source: Sci Rep. 2021 Sep 29;11:19308. doi: 10.1038/s41598-021-98211-5 (PMC8481482; doi:10.1038/s41598-021-98211-5)
Supplement: Supplementary file 1 — Supplementary Information. [file 41598_2021_98211_MOESM1_ESM.pdf]

Supplemental Material for "A random-walk-based epidemiological model"

Andrew Chu,[1] Greg Huber,[1] Aaron McGeever,[1] Boris Veytsman,[2] and David Yllanes[1]

[1] *Chan Zuckerberg Biohub, 499 Illinois Street, San Francisco, CA 94158, USA*

[2] *Chan Zuckerberg Initiative, 601 Marshall Street, Redwood City, CA 94063, USA*

I. COMPUTATION OF THE PHASE DIAGRAM

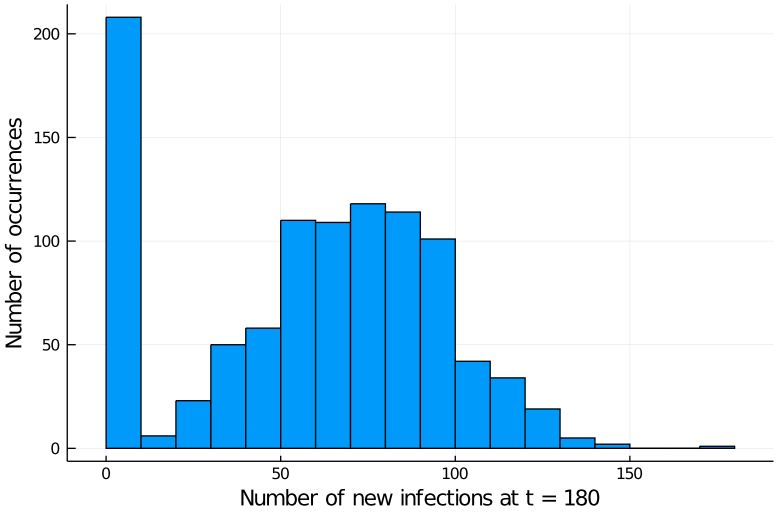

FIG. S1: Histogram of epidemic growth rate across several realizations of the outbreak for $p = 0.5$ and $\tau = 4$. Note that 1000 independent runs are used here to provide additional detail for the reader, but the model uses 100 runs for computational efficiency.

The data from running 100 realizations for each (p, τ) pair was used to produce a histogram of the growth rate (defined as the number of new infections) at the final time step, as shown in Figure S1. The final time step is chosen by the following function:

$$t(\tau) = \begin{cases} 200 & \text{if } \tau < 20 \\ 10\tau & \text{if } \tau \geq 20 \end{cases} \tag{1}$$

This sliding criterion adjusts for outbreaks with longer τ, i.e., when infective agents have longer lifespans. Notably, the histogram exhibits a large spike at zero new infections, which represents the fact that a significant fraction of outbreaks will die out quickly, since, due to the intrinsic stochasticity of our model, not every infection reaches epidemic-level proportions. From this histogram, a cutoff of 10 new infections at the final time step is used as a proxy for determining whether an outbreak has died out.

II. ATTACK RATE AND RADIUS OF GYRATION ACROSS THE PHASE BOUNDARY

In this section, we explore two alternative metrics to characterize the incidence and growth of an epidemic. First, the *attack rate* is generally defined as the proportion of exposed susceptibles that have been infected. Given the set of infected sites, I, the set of removed sites, R, and the set of visited sites (which includes infected and removed ones), V, we may define the attack rate in the context of our lattice-based model. Using $|X|$ to represent the cardinality of set X, attack rate is calculated as

$$\text{attack rate} = \frac{|I| + |R|}{|V|} \,. \tag{2}$$

In our spatial model, we can also study the growth of the affected area. To this end, a simple and intuitive metric is the radius of gyration $\mathcal{R}_\mathrm{g}$ of the set of visited sites. If $\mathbf{r}_i$ is the position of the i-th visited site, then

$$\mathcal{R}_g^2 = \frac{1}{|V|} \sum_{i \in V} \left(\mathbf{r}_i - \mathbf{r}_\mathrm{CM}\right)^2 , \qquad \mathbf{r}_\mathrm{CM} = \frac{1}{|V|} \sum_{i \in V} \mathbf{r}_i. \tag{3}$$

The attack rate and radius of gyration for selected p and τ values along two cross-sections of the phase diagram near the phase boundary were plotted and are shown in Figure SS2. These two transects of the phase diagram were chosen to capture the effects of crossing the boundary from different positions. For each transect, 10 different p, τ pairs are sampled and then the attack rate and radius of gyration are calculated for each pair. These computations are averaged from 1000 realizations of the model for 800 time steps. For the fixed-τ interval, $\tau = 50$ and p ranges from 0.01 to 0.10 (in increments of 0.01). For the fixed-p interval, $p = 0.4$ and τ ranges from 1 to 10 (in increments of 1).

Figures S2(b) and S2(d) show how the attack rate increases with p and τ. Notice that for very small values of τ, below the phase boundary, the attack rate can decrease with time. This is because we normalize by the number of visited sites, which is always one at start but increases with time. The attack rate of eq. (2), hence, is initially equal to p but can decrease for dying epidemics as more sites are visited but not infected.

Figures S2(c) and S2(e) clearly show the transition between the extinction regime (where $\mathcal{R}_\mathrm{g}$ plateaus) and the regime of indefinite spreading. The latter, for fixed $\tau = 50$ and small p, has a more punctuated change after $t = \tau = 50$, when several infections begin to plateau and die out. This behavior is explained by the low p values, which cause most outbreaks to stop growing after the lifetime of the initial walker expires at $t = 50$.

III. SUPPLEMENTARY FIGURES

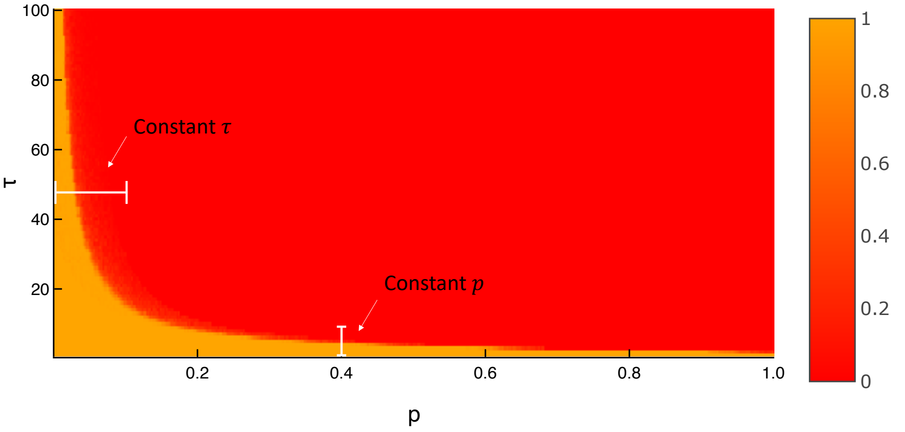

(a)

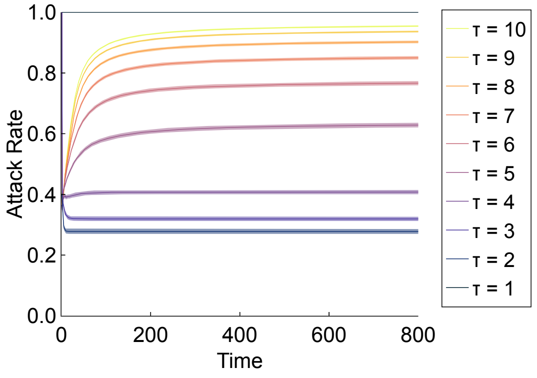

(b)

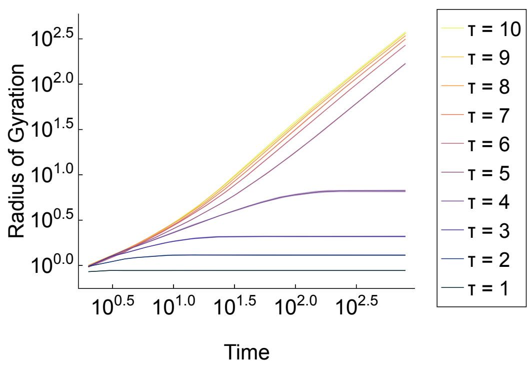

(c)

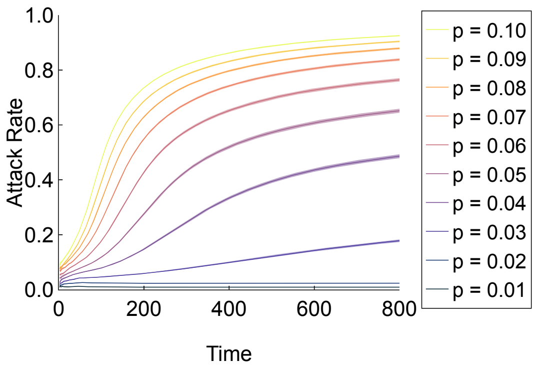

(d)

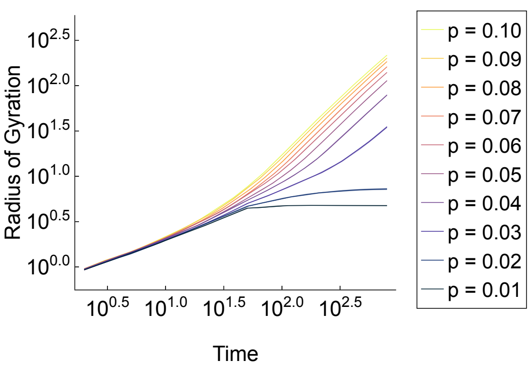

(e)

FIG. S2: Study of the radius of gyration of the set of infected sites and of the attack rate performed across two transects of the phase diagram. 2(a) Location of the two transects in the phase diagram that are examined in detail. 1000 outbreaks of 800 time steps are simulated for each p and τ combination and the attack rate and radius of gyration are calculated. Note that both of the studied transects cross the boundary between phases. Solving equation (4) in the main text for $R_0 = 1.39$, we see that the boundary for $\tau = 50$ is at $p \approx 0.0283$. For $p = 0.4$, $\tau < 4$ are in the extinction regime. 2(b) The attack rate for the fixed-p interval. 2(c) The radius of gyration for the fixed-p interval. 2(d) The attack rate for the fixed-τ interval ($p < 0.03$ are in the extinction regime). 2(e) The radius of gyration for the fixed-τ interval.

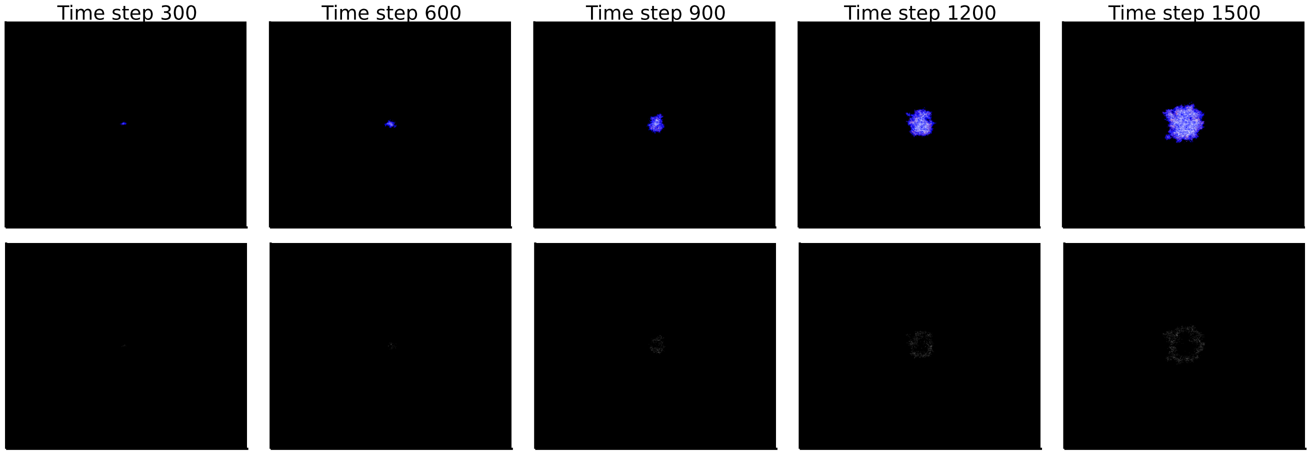

FIG. S3: *Top:* Snapshots of a sample simulation with $\tau = 50$ and $p = 0.03$, up to 1500 time steps. We show the total outbreak, with the color scheme of Figure 3 in the main text (red: infected sites, white: removed sites, blue: visited but not removed sites). *Bottom:* For the same runs, we show only the set of currently infected sites (in white for greater contrast), to show the width and fractal nature of the boundary. The depicted boxes have 1400x1400 sites.

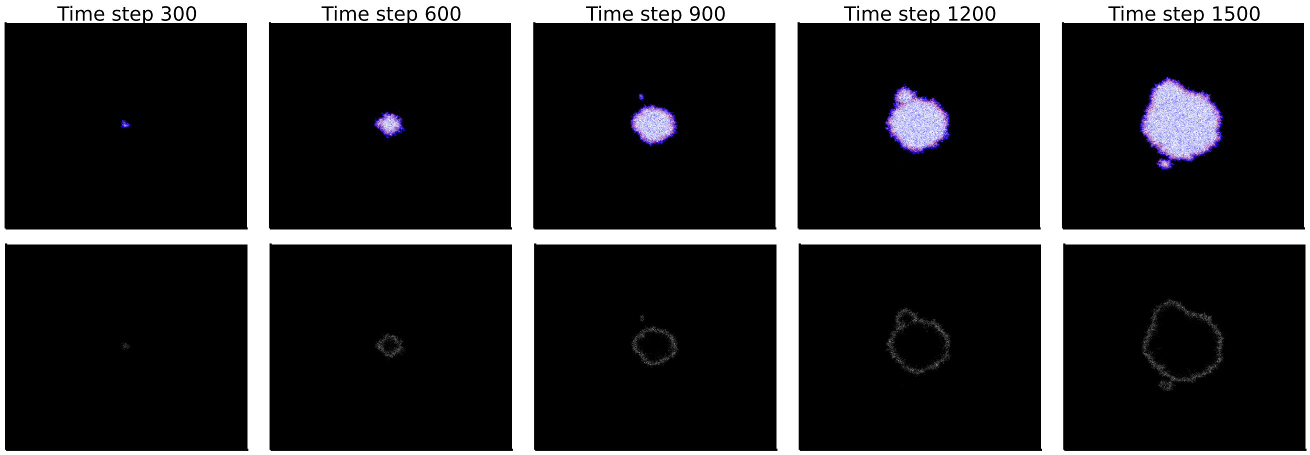

FIG. S4: As in Figure S3, but for $p = 0.04$.

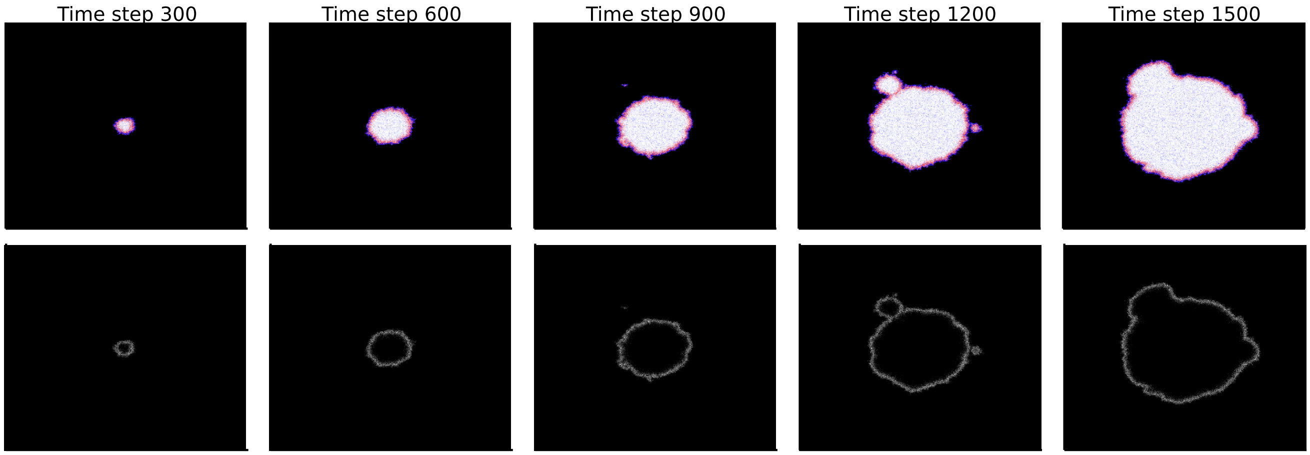

FIG. S5: As in Figure S3, but for $p = 0.06$.

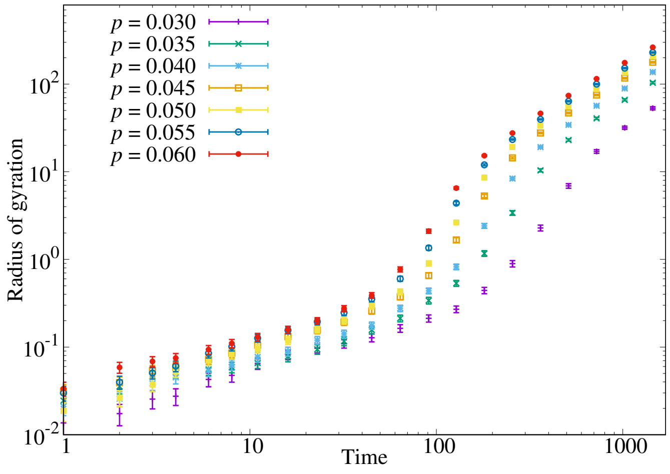

FIG. S6: Time evolution of the radius of gyration for $\tau = 50$ and the same p values considered in the main text. For long times, a power-law growth is approached.

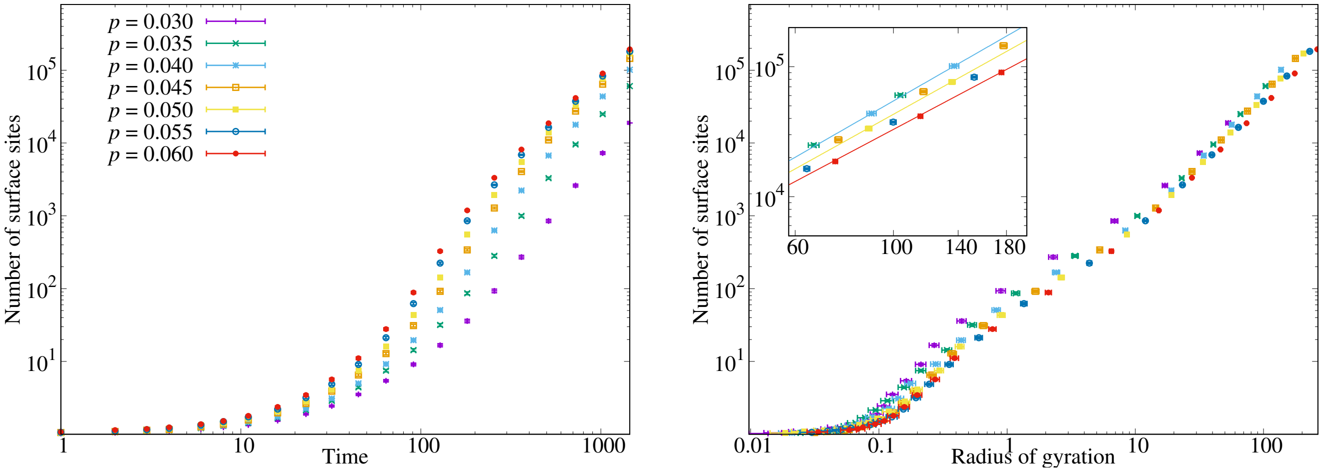

FIG. S7: Growth of the number of surface sites in the cluster of removed sites. As for the total mass of the cluster (plotted in Figure 6 of the main text), a power-law growth is observed when plotted against the radius of gyration. Unlike, Figure 6, however, the curves for different p do not collapse (but those closest to the critical region, i.e., $p \leq 0.04$, do). We show power-law fits for $p = 0.04$, 0.05 and $p = 0.06$.

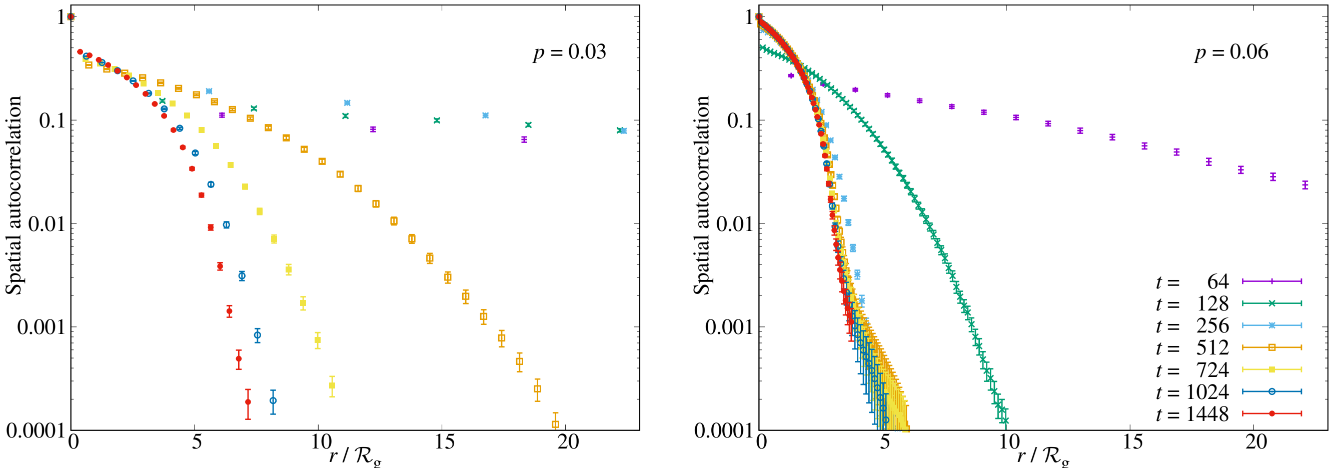

FIG. S8: Spatial autocorrelation of the cluster of removed sites, as in the bottom panels of Figure 6 in the main text, for $p = 0.03$ and $p = 0.06$, in both cases with $\tau = 50$. As we move deeper into the spreading phase, the enveloping curve is approached for much shorter times.
